# Sex differences in adverse events in Medicare individuals ≥ 66 years of age post glioblastoma treatment

**DOI:** 10.1007/s11060-024-04652-z

**Published:** 2024-04-02

**Authors:** Mantas Dmukauskas, Gino Cioffi, Kristin A. Waite, Andrew E. Sloan, Corey Neff, Mackenzie Price, Quinn T. Ostrom, Jill S. Barnholtz-Sloan

**Affiliations:** 1 Trans Divisional Research Program, Division of Cancer Epidemiology and Genetics, National Cancer Institute, Bethesda, MD USA; 2 Neuroscience Service Line and Piedmont Brain Tumor Center, Piedmont Health, Atlanta, GA USA; 3 Department of Neurosurgery, Duke University School of Medicine, Durham, NC USA; 4 The Preston Robert Tisch Brain Tumor Center, Duke University School of Medicine, Durham, NC USA; 5 Center for Biomedical Informatics and Information Technology, National Cancer Institute, Shady Grove Campus 9609 Medical Center Dr, 20850 Rockville, MD USA

**Keywords:** Glioma, Adverse events, Sex difference, SEER-Medicare

## Abstract

**Purpose:**

Glioblastoma (GB) is the most common primary malignant brain tumor with the highest incidence occurring in older adults with a median age at diagnosis of 64 years old. While treatment often improves survival it brings toxicities and adverse events (AE). Here we identify sex differences in treatment patterns and AE in individuals ≥ 66 years at diagnosis with GB.

**Methods:**

Using the SEER-Medicare dataset sex differences in adverse events were assessed using multivariable logistic regression performed to calculate the male/female odds ratio (M/F OR) and 95% confidence intervals [95% CI] of experiencing an AE adjusted for demographic variables and Elixhauser comorbidity score.

**Results:**

Males with GB were more likely to receive standard of care (SOC; Surgery with concurrent radio-chemotherapy) [20%] compared to females [17%], whereas females were more likely to receive no treatment [26%] compared to males [21%]. Females with GB receiving SOC were more likely to develop gastrointestinal disorders (M/F OR = 0.76; 95% CI,0.64–0.91, *p* = 0.002) or blood and lymphatic system disorders (M/F OR = 0.79; 95% CI,0.66–0.95, *p* = 0.012). Males with GB receiving SOC were more likely to develop cardiac disorders (M/F OR = 1.21; 95% CI,1.02–1.44, *p* = 0.029) and renal disorders (M/F OR = 1.65; 95% CI,1.37–2.01, *p* < 0.001).

**Conclusions:**

Sex differences for individuals, 66 years and older, diagnosed with GB exist in treatment received and adverse events developed across different treatment modalities.

**Supplementary Information:**

The online version contains supplementary material available at 10.1007/s11060-024-04652-z.

## Introduction

Glioblastoma (GB) is the most common type of glioma accounting for approximately 60% of all gliomas and 51% of all malignant brain tumors [1]. In the United States (US), the incidence rate for GB is 5 per 100,000 persons, with a 5-year relative survival of less than 7% [1]. In adults, the risk of developing a GB increases with age, with the median age at diagnosis of a GB being 64 years old [2]. Males have been shown to have increased GB risk and worse GB survival rates when compared to females [1, 3]. 

Despite aggressive multi-modal therapies, GB remains uniformly lethal. Progression-free survival is dictated by response to extent of surgical resection and radiation and chemotherapy. Current standard of care (SOC) for GB was established nearly two decades ago (i.e. the Stupp protocol) and confers only a modest increase in overall survival [4–6]. When first established, the SOC protocol for GB was limited to individuals under 70 year-old [4] and there is still a limited number of clinical trials involving elderly individuals [7]. The lack of rigorous information on clinical outcomes in the aging population often leads to treatment strategies based on physician and/or individual preference, taking into consideration prognostic survival benefits as well as potential toxicities and risk of adverse events (AE) [8–11]. AEs can range from mild disorders to life-threatening conditions, including increased risk of neurological deficiency after surgical intervention, fibrosis after radiation treatment, and psychiatric disorders and cardiovascular complications following chemotherapy [12–16]. Multimodal treatment approaches increase the risk of tissue toxicities compared to a single treatment approach [17]. 

Recent work has suggested the presence of a sex-bias in the development of AE in individuals receiving cancer treatment [18–20]. However, the impact of various treatment modalities on the development of AE in individuals with GB has not been closely examined. We aim to fill this gap in knowledge by utilizing the population-based data available from the Surveillance, Epidemiology and End Results (SEER) Program-Medicare dataset to investigate sex differences in AEs based on treatment modalities in individuals with GB.

## Methods

### Study design and setting

Individuals diagnosed with GB were obtained from the SEER-Medicare dataset, a population based data registry collecting demographic, clinical, and cause of death information from individuals with cancer covering about 28% of the US population [21]. Medicare is a US federally funded health insurance program primarily involving individuals that are 65 and older. Medicare claims are insurance billing data for treatment or diagnostic procedures. Medicare claims data are linked with the SEER dataset, through an agreement with the National Cancer Institute (NCI) and the Center for Medicare Services (CMS), providing additional information on older adults with cancer. This study was conducted at the NCI and was determined to be human subjects exempt by the National Institute of Health (NIH) Office of Human Subjects Research.

### Claims data

The following claims files from SEER-Medicare were utilized in this study: Durable Medical Equipment (DME), Carrier Claims (NCH), Outpatient Claims, Medicare Provider Analysis and Review (MedPAR), and Medicare Part D Data. DME and NCH files contained data on physician-performed procedures and visits while the Outpatient Claims file contained outpatient procedures. The MedPAR file contained data on hospitalization and the Medicare Part D data contained information on prescription drugs. AE were collected from DME, NCH, Outpatient, and MedPAR data files.

### Inclusion/exclusion criteria

A total of 12,165 individuals diagnosed between 2004 and 2017 were selected for this study. Individuals were restricted to those aged 66 years and above to ensure a full year of claims data prior to the diagnosis for evaluating their comorbidities. Individuals with GB were selected using *International Classification of Disease for Oncology, Third Edition (ICD-O-3)* histopathology codes (9440, 9441, 9442/ 3, 9445) consistent with the reporting of Central Brain Tumor Registry of the United States (CBTRUS) [22]. Individuals were excluded if they were either enrolled in a Health Maintenance Organization (HMO) or if they lacked continuous Medicare part A and part B enrollment for 1 year prior to the date of diagnosis through the end of adverse event observation time (6–12 months after diagnosis) or date of death/last follow up, whichever occurred first.

### Data collected

First-line cancer treatment information was collected from claims data using the following categories: surgery, radiation, and Temozolomide. Temozolomide is the most common GB chemotherapy agent and a key component of the current SOC for GB. Treatment modalities were identified in the claims data using ICD9/10 procedure codes, Healthcare Common Procedure Coding System (HCPCS) codes, and Current Procedural Terminology (CPT) codes (Supplemental Table 1). The SEER registry contains reliable information regarding individual treatment by both surgery and radiation [23, 24]. Thus, the SEER database was utilized to identify surgery and radiation treatment patterns for individuals lacking treatment data from the claims dataset. This approach maximized sample size for this rare tumor type. Individuals with any treatment modality in either Medicare claims data or surgery and radiation data in the SEER dataset within 6 months following diagnosis were classified as having received treatment. Individuals whose first-line therapy started more than 6 months after diagnosis were classified as receiving no treatment. To assess the impact of various treatment modalities, individuals with GB were analyzed by specific treatment modality as follows: SOC (surgery, radiation, and Temozolomide), surgery alone, Temozolomide alone, radiation alone, and any other pairwise combination of surgery, Temozolomide, and radiation.

Demographic data assessed included sex (male, female), ethnicity (Hispanic, non-Hispanic), age at diagnosis, and race (White, Black, “Other”). The race category “Other”, consisted of Native American, Alaskan Indian, Asian and Pacific Islander, combined due to the insufficient sample size to assess these groups separately. The Elixhauser comorbidity score was calculated with R package ‘Comorbidity 1.0.0’, using claims data and including all comorbidities 1 year prior to the date of GB diagnosis. Comorbidity scores were grouped into the following categories: 0–3, 4–6, 7–9, and > 9 [25]. 

### Adverse events (AE)

AEs categories were identified using the standardized classification in the common terminology criteria of adverse event (CTCAE) designed to identify AEs in clinical trials. The CTCAE uses the Medical Dictionary for Regulatory Activities (MedDRA) code system to identify the AEs (Supplemental Table 2). MedDRA AE codes were converted to Systematized Nomenclature of Medicine (SNOMED) codes using MedDRA mapping tool. The resulting SNOMED codes were then converted to ICD10 codes. Further, we analyzed individual AEs that occurred between 0 and 6 months after the start of any treatment or, for individuals that received no treatment, after the diagnosis. AEs that occurred between the date of diagnosis and the start of treatment were excluded from the study. AE groups that were either not relevant to individuals over the age of 66, specific to reproductive organs, specific to congenital disorders, or had a small, limited number of individuals were excluded in the analysis. These included congenital, familial, and genetic disorders; immune system disorders; hepatobiliary disorders; injury, poisoning, and procedural complications; reproductive system and breast disorders; investigations; and pregnancy, puerperium and perinatal conditions.

AE categories analyzed include blood and lymphatic system disorders; cardiac disorders; ear and labyrinth disorders; endocrine disorders; eye disorders; gastrointestinal disorders; general disorders and administration site conditions (conditions of a general kind that result from or treatment of a disease); infections and infestations; metabolism and nutrition disorders; musculoskeletal and connective tissue disorders; neoplasms benign, malignant and unspecified (including cysts and polyps); nervous system disorders; psychiatric; renal and urinary disorders; respiratory, thoracic and mediastinal disorders; skin and subcutaneous tissue disorders; and vascular disorders. All AEs were treated as binary outcomes (yes/no).

### Statistical analysis

All statistical analyses were performed using R 4.1 software. Descriptive statistics, stratified by sex, were used to assess differences in the sex distribution of demographic and clinical factors. Groups of individuals with each treatment modality combination were analyzed separately. Counts are suppressed when fewer than 11 cases were reported within a cell, or where the inclusion of the count would allow for back-calculation of suppressed values. The suppressed cases are included in the total counts for a given category.

Continuous data were assessed using t-test and categorical data were assessed with either Fisher’s exact test or Pearson’s Chi-squared test where appropriate. Multivariable logistic regression, adjusted for demographic variables and Elixhauser comorbidity score, was performed to calculate the male/female odds ratio (M/F OR) and 95% confidence intervals [95% CI] of experiencing an AE. Separate multivariable logistic regression models were performed for each AE category, among each treatment modality. All M/F ORs are reported as the odds of males experiencing an adverse event compared to females. Pearson correlation was used to assess the relationship between individual AEs to elucidate if the presence of certain events were driving the chance of experiencing other events. *P* values less than 0.05 were considered statistically significant.

## Results

### Study population and individual characteristics

**Fig. 1:**
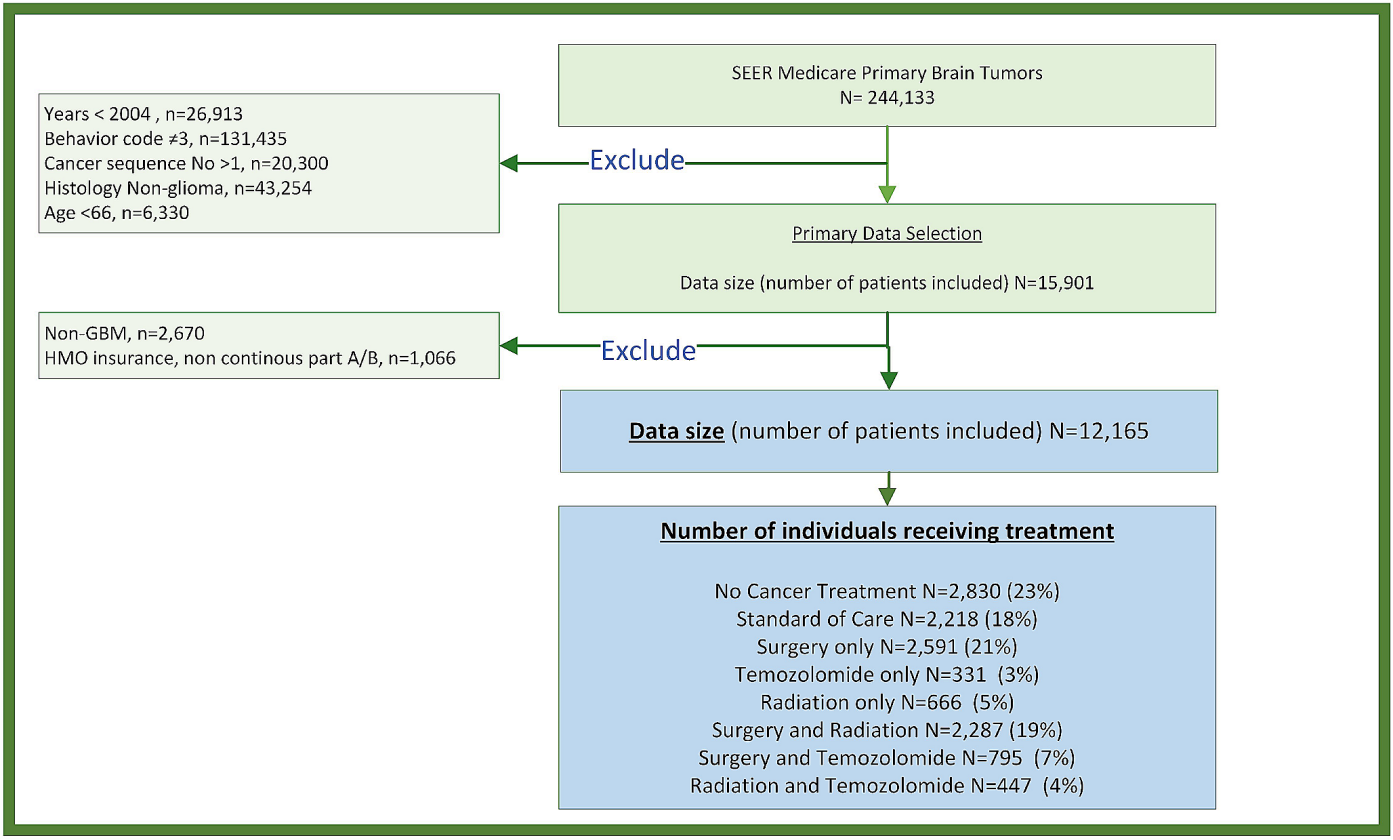
Data selection and exclusion flow chart: The number of individuals selected for the study are summarized. The schema provides the description and number of individuals excluded from the study. Numbers of individuals with glioblastoma and their treatment modalities are summarized

The SEER-Medicare dataset contained 244,133 individuals diagnosed with brain tumors. A total of 231,968 records were excluded for one or more reasons: (1) a histopathology other than GB, (2) brain tumor diagnosis before 2004, (3) a diagnosis of non-first sequence brain tumor, (4) age of diagnosis < 66 years, (5) and a record of HMO or non-continuous part A and part B enrollment (Fig. 1). After exclusion there were 12,165 individuals diagnosed with GB, consisting of 6,304 males (52%) and 5,861 females (48%).

Individuals diagnosed with GB were predominately White (92%, male to female ratio (m/f) 93%/92%) and non-Hispanic (93%). There was a significant sex difference in race (*p* < 0.001), but not in ethnicity (*p* = 0.3). The average age of an individual diagnosed with GB, in this Medicare population, was 75 years old. There was a significant sex difference in age of diagnosis (*p* < 0.001, m/f 75/76 years old) and Elixhauser score (*p* = 0.013) with more males having lower comorbidity score at diagnosis compared to females (0–3 m/f 48%/46%; 4–6 m/f 26%/25%; 7–9 m/f 16%/16%; >9 m/f 11%/13%). The average age of individuals with GB receiving SOC was 73 (m/f 73/73, *p* = 0.3) years old whereas the average age in those receiving no treatment was 79 (m/f 78/80, *p* < 0.001) years old. Only 2,218 (18%) individuals received SOC (surgery with concurrent radio-chemotherapy), while most individuals (59%) received other treatment regiments (surgery only, Temozolomide only, radiation only, and any other combination of surgery, Temozolomide, and radiation), and 2,830 (23%) received no cancer related treatment (Table 1). There was a significant sex difference in treatment modality received by individuals with GB: males were more likely to receive SOC compared to females (*n* = 1,238 [20%] males and 980 [17%] females, *p* < 0.001). A significant sex difference was observed in individuals receiving no treatment (1,307 males [21%], 1,523 females [26%], *p* < 0.001).

**Table 1:** Glioblastoma descriptive table. Descriptive statistics of demographic and clinical factors of individuals with glioblastoma is shown. The data is stratified by treatment modality and sex

	No Treatment, N=2,830 (23%)^1^			Standard of Care, *N* = 2,218 (18%)^1^			Surgery and Radiation, *N* = 2,287 (19%)^1^			Surgery and Temozolomide, *N* = 795 (6.5%)^1^			Radiation and Temozolomide, *N* = 447 (3.7%)^1^			Surgery Only, *N* = 2,591 (21%)^1^			Radiation Only, *N* = 666 (5.5%)^1^			Temozolomide Only, *N* = 331 (2.7%)^1^		
	Female, N = 1523 (54%)^1^	Male, N = 1307 (46%)^1^	*p*-value^2^	Female, N = 980 (44%)^1^	Male, N = 1238 (56%)^1^	*p*-value^2^	Female, N = 1081 (47%)^1^	Male, N = 1206 (53%)^1^	*p*-value^2^	Female, N = 372 (47%)^1^	Male, N = 423 (53%)^1^	*p*-value^2^	Female, N = 210 (47%)^1^	Male, N = 237 (53%)^1^	*p*-value^3^	Female, N = 1221 (47%)^1^	Male, N = 1370 (53%)^1^	*p*-value^2^	Female, N = 323 (48%)^1^	Male, N = 343 (52%)^1^	*p*-value^2^	Female, N = 151 (46%)^1^	Male, N = 180 (54%)^1^	*p*-value^3^
Race			0.7			0.2			0.006			0.2			0.2			0.2			0.3			0.15
White	1,396 (92%)	1,203 (92%)		918 (94%)	1,165 (94%)		965 (89%)	1,103 (91%)		340 (91%)	400 (95%)		191 (91%)	226 (95%)		1,140 (93%)	1,278 (93%)		291 (90%)	297 (87%)		141 (93%)	174 (97%)	
Black	74 (4.9%)	56 (4.3%)		33 (3.4%)	28 (2.3%)		79 (7.3%)	52 (4.3%)		21 (5.6%)	-		-	-		59 (4.8%)	55 (4.0%)		21 (6.5%)	26 (7.6%)		-	-	
Other	53 (3.5%)	48 (3.7%)		29 (3.0%)	45 (3.6%)		37 (3.4%)	51 (4.2%)		11 (3.0%)	-		-	-		22 (1.8%)	37 (2.7%)		11 (3.4%)	20 (5.8%)		-	-	
Ethnicity			0.3			0.062			0.7			0.5			0.8			0.2			0.4			0.066
Non-Hispanic	1,430 (94%)	1,214 (93%)		898 (92%)	1,160 (94%)		988 (91%)	1,108 (92%)		346 (93%)	398 (94%)		199 (95%)	223 (94%)		1,124 (92%)	1,279 (93%)		304 (94%)	317 (92%)		-	-	
Hispanic	93 (6.1%)	93 (7.1%)		82 (8.4%)	78 (6.3%)		93 (8.6%)	98 (8.1%)		26 (7.0%)	25 (5.9%)		11 (5.2%)	14 (5.9%)		97 (7.9%)	91 (6.6%)		19 (5.9%)	26 (7.6%)		-	-	
Elixhauser Comorbidity Score			0.034			0.3			0.8			0.2			0.066			0.8			0.5			0.8
0–3	655 (43%)	632 (48%)		389 (40%)	480 (39%)		661 (61%)	741 (61%)		133 (36%)	168 (40%)		65 (31%)	93 (39%)		564 (46%)	653 (48%)		175 (54%)	180 (52%)		54 (36%)	60 (33%)	
4–6	347 (23%)	282 (22%)		309 (32%)	422 (34%)		220 (20%)	257 (21%)		120 (32%)	137 (32%)		81 (39%)	68 (29%)		300 (25%)	329 (24%)		59 (18%)	72 (21%)		43 (28%)	49 (27%)	
7–9	275 (18%)	205 (16%)		168 (17%)	219 (18%)		123 (11%)	132 (11%)		75 (20%)	85 (20%)		37 (18%)	52 (22%)		190 (16%)	215 (16%)		45 (14%)	54 (16%)		32 (21%)	37 (21%)	
> 9	246 (16%)	188 (14%)		114 (12%)	117 (9.5%)		77 (7.1%)	76 (6.3%)		44 (12%)	33 (7.8%)		27 (13%)	24 (10%)		167 (14%)	173 (13%)		44 (14%)	37 (11%)		22 (15%)	34 (19%)	
Age	80 (7)	78 (8)	< 0.001	73.0 (5.3)	72.7 (5.1)	0.3	73.6 (5.7)	73.3 (5.4)	0.2	73.4 (5.3)	73.0 (5.5)	0.4	74.7 (5.8)	74.7 (6.0)	0.9	76.0 (6.2)	75.5 (6.1)	0.034	77 (7)	76 (7)	0.2	74 (6)	75 (6)	0.4

^1^n (%); Mean (SD)

^2^Pearson's Chi-squared test; Welch Two Sample t-test

^3^Fisher's exact test; Pearson's Chi-squared test; Welch Two Sample t-test

### Adverse events (AE)

**Fig. 2:**
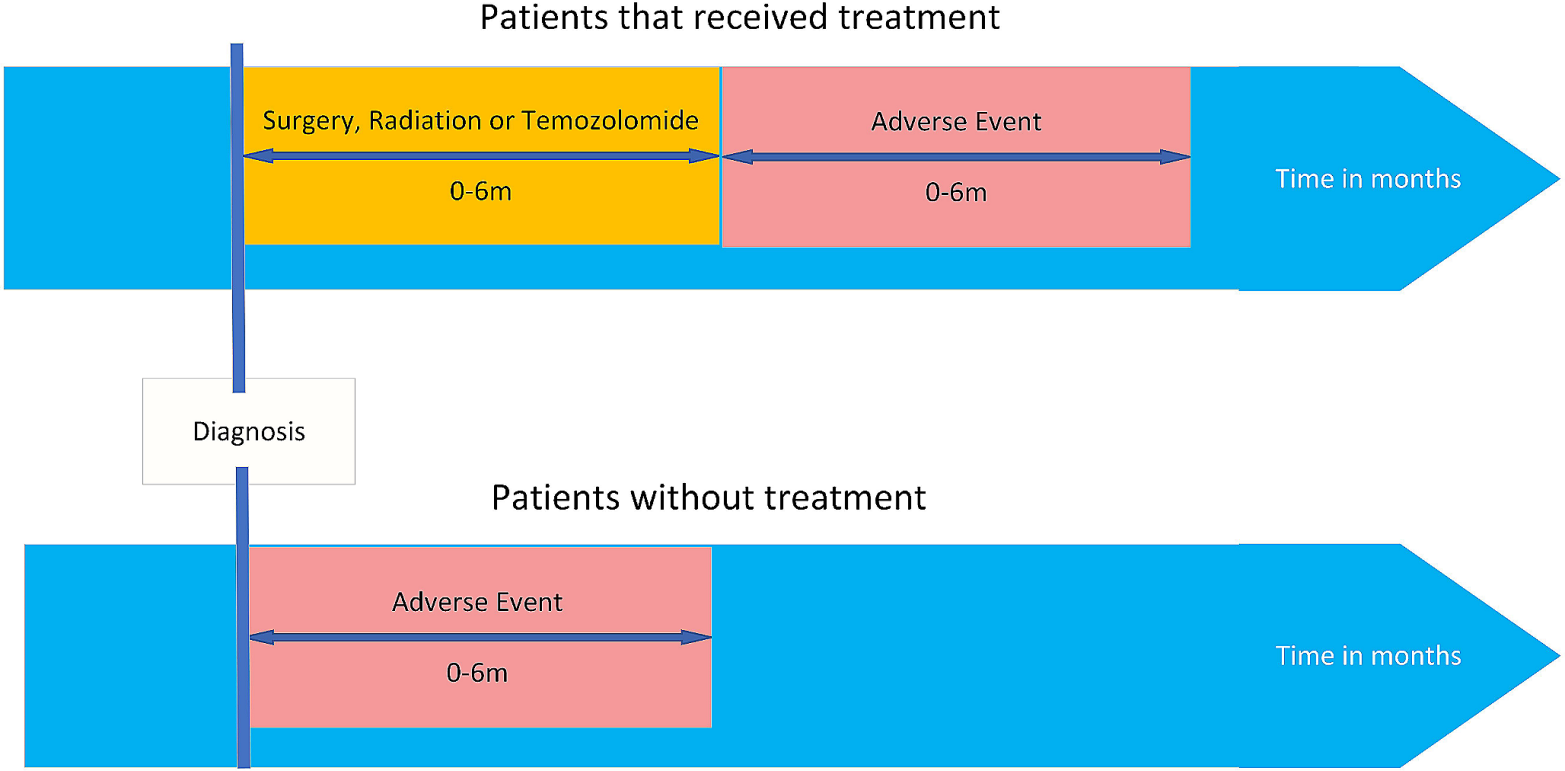
Schema of treatment and adverse event selection criteria: The schema depicts the timeline used for adverse event selection for this study

Results for individual AEs that occurred between 0 and 6 months after the start of treatment or, for individuals that received no treatment, after the diagnosis date (Fig. 2) are shown. Regardless of treatment modality, both males and females individually experienced on average 7 unique types of AEs. Nervous system disorders were the most common (78%) followed by vascular disorders (66%) (Table 2). All pairs of AEs had a correlation below 0.5 with the highest occurring between nervous system disorders and general and administration site conditions (*r*^2^ = 0.43) (Supplementary Fig. 1).

**Table 2:** Glioblastoma individuals experiencing adverse events post treatment. Descriptive statistics of adverse events experienced by individuals with glioblastoma. The data is stratified by treatment modality and sex

No Treatment, *N* = 2830 (23%)^1^				Standard of Care, *N* = 2218 (18%)^1^			Surgery and Radiation, *N* = 2287 (19%)^1^			Surgery and Temozolomide, *N* = 795 (6.5%)^1^			Radiation and Temozolomide, *N* = 447 (3.7%)^1^			Surgery Only, *N* = 2591 (21%)^1^			Radiation Only, *N* = 666 (5.5%)^1^			Temozolomide Only, *N* = 331 (2.7%)^1^		
	Female, N = 1523 (54%)^1^	Male, N = 1307 (46%)^1^	*p*-value^2^	Female, N = 980 (44%)^1^	Male, N = 1238 (56%)^1^	*p*-value^2^	Female, N = 1081 (47%)^1^	Male, N = 1206 (53%)^1^	*p*-value^2^	Female, N = 372 (47%)^1^	Male, N = 423 (53%)^1^	*p*-value^2^	Female, N = 210 (47%)^1^	Male, N = 237 (53%)^1^	*p*-value^2^	Female, N = 1221 (47%)^1^	Male, N = 1370 (53%)^1^	*p*-value^2^	Female, N = 323 (48%)^1^	Male, N = 343 (52%)^1^	*p*-value^2^	Female, N = 151 (46%)^1^	Male, N = 180 (54%)^1^	*p*-value^2^
Blood and lymphatic system	273 (18%)	255 (20%)	0.3	356 (36%)	382 (31%)	0.007	326 (30%)	366 (30%)	> 0.9	143 (38%)	140 (33%)	0.12	68 (32%)	53 (22%)	0.017	307 (25%)	318 (23%)	0.3	87 (27%)	81 (24%)	0.3	44 (29%)	45 (25%)	0.4
Cardiac	575 (38%)	597 (46%)	< 0.001	436 (44%)	602 (49%)	0.052	367 (34%)	494 (41%)	< 0.001	190 (51%)	225 (53%)	0.6	86 (41%)	112 (47%)	0.2	467 (38%)	597 (44%)	0.006	135 (42%)	151 (44%)	0.6	60 (40%)	80 (44%)	0.4
Ear and labyrinth	111 (7.3%)	94 (7.2%)	> 0.9	126 (13%)	168 (14%)	0.6	76 (7.0%)	107 (8.9%)	0.11	56 (15%)	64 (15%)	> 0.9	15 (7.1%)	20 (8.4%)	0.6	66 (5.4%)	114 (8.3%)	0.004	29 (9.0%)	32 (9.3%)	0.9	15 (9.9%)	21 (12%)	0.6
Endocrine	342 (22%)	134 (10%)	< 0.001	226 (23%)	175 (14%)	< 0.001	265 (25%)	139 (12%)	< 0.001	93 (25%)	58 (14%)	< 0.001	40 (19%)	20 (8.4%)	0.001	242 (20%)	125 (9.1%)	< 0.001	62 (19%)	52 (15%)	0.2	32 (21%)	19 (11%)	0.008
Eye	104 (6.8%)	90 (6.9%)	> 0.9	105 (11%)	122 (9.9%)	0.5	89 (8.2%)	102 (8.5%)	0.8	38 (10%)	42 (9.9%)	0.9	13 (6.2%)	14 (5.9%)	> 0.9	86 (7.0%)	79 (5.8%)	0.2	25 (7.7%)	23 (6.7%)	0.6	12 (7.9%)	11 (6.1%)	0.5
Gastrointestinal	660 (43%)	569 (44%)	> 0.9	629 (64%)	715 (58%)	0.002	578 (53%)	582 (48%)	0.013	215 (58%)	282 (67%)	0.010	128 (61%)	131 (55%)	0.2	590 (48%)	639 (47%)	0.4	173 (54%)	181 (53%)	0.8	85 (56%)	98 (54%)	0.7
General and administration site conditions	674 (44%)	585 (45%)	0.8	763 (78%)	909 (73%)	0.016	553 (51%)	651 (54%)	0.2	278 (75%)	331 (78%)	0.2	149 (71%)	157 (66%)	0.3	651 (53%)	710 (52%)	0.4	188 (58%)	191 (56%)	0.5	86 (57%)	113 (63%)	0.3
Infections and infestations	584 (38%)	387 (30%)	< 0.001	500 (51%)	528 (43%)	< 0.001	465 (43%)	417 (35%)	< 0.001	200 (54%)	198 (47%)	0.050	103 (49%)	105 (44%)	0.3	511 (42%)	522 (38%)	0.052	156 (48%)	122 (36%)	< 0.001	71 (47%)	72 (40%)	0.2
Metabolism and nutrition	799 (52%)	741 (57%)	0.024	664 (68%)	843 (68%)	0.9	654 (60%)	772 (64%)	0.083	246 (66%)	301 (71%)	0.13	132 (63%)	140 (59%)	0.4	703 (58%)	791 (58%)	> 0.9	197 (61%)	219 (64%)	0.4	75 (50%)	104 (58%)	0.14
Musculoskeletal and connective tissue	642 (42%)	426 (33%)	< 0.001	624 (64%)	684 (55%)	< 0.001	494 (46%)	490 (41%)	0.015	219 (59%)	227 (54%)	0.14	113 (54%)	123 (52%)	0.7	529 (43%)	511 (37%)	0.002	152 (47%)	153 (45%)	0.5	69 (46%)	78 (43%)	0.7
Neoplasms benign malignant and unspecified incl cysts and polyps	718 (47%)	573 (44%)	0.079	712 (73%)	889 (72%)	0.7	495 (46%)	579 (48%)	0.3	298 (80%)	328 (78%)	0.4	100 (48%)	90 (38%)	0.040	674 (55%)	720 (53%)	0.2	148 (46%)	162 (47%)	0.7	74 (49%)	75 (42%)	0.2
Nervous system	1,154 (76%)	963 (74%)	0.2	851 (87%)	1,064 (86%)	0.5	793 (73%)	908 (75%)	0.3	328 (88%)	386 (91%)	0.2	165 (79%)	186 (78%)	> 0.9	900 (74%)	1,047 (76%)	0.11	257 (80%)	261 (76%)	0.3	104 (69%)	132 (73%)	0.4
Psychiatric	572 (38%)	406 (31%)	< 0.001	444 (45%)	485 (39%)	0.004	420 (39%)	445 (37%)	0.3	179 (48%)	186 (44%)	0.2	91 (43%)	90 (38%)	0.2	456 (37%)	494 (36%)	0.5	128 (40%)	121 (35%)	0.2	53 (35%)	63 (35%)	> 0.9
Renal and urinary	239 (16%)	271 (21%)	< 0.001	231 (24%)	415 (34%)	< 0.001	219 (20%)	332 (28%)	< 0.001	94 (25%)	162 (38%)	< 0.001	47 (22%)	75 (32%)	0.028	223 (18%)	351 (26%)	< 0.001	65 (20%)	97 (28%)	0.014	36 (24%)	49 (27%)	0.5
Respiratory thoracic and mediastinal	547 (36%)	548 (42%)	0.001	548 (56%)	695 (56%)	> 0.9	425 (39%)	556 (46%)	0.001	199 (53%)	266 (63%)	0.007	101 (48%)	127 (54%)	0.2	519 (43%)	704 (51%)	< 0.001	140 (43%)	167 (49%)	0.2	71 (47%)	91 (51%)	0.5
Skin and subcutaneous tissue	98 (6.4%)	94 (7.2%)	0.4	209 (21%)	279 (23%)	0.5	145 (13%)	176 (15%)	0.4	63 (17%)	86 (20%)	0.2	44 (21%)	29 (12%)	0.013	113 (9.3%)	122 (8.9%)	0.8	32 (9.9%)	34 (9.9%)	> 0.9	28 (19%)	35 (19%)	0.8
Vascular	985 (65%)	803 (61%)	0.075	710 (72%)	913 (74%)	0.5	690 (64%)	768 (64%)	> 0.9	279 (75%)	313 (74%)	0.7	130 (62%)	144 (61%)	0.8	785 (64%)	856 (62%)	0.3	224 (69%)	216 (63%)	0.082	89 (59%)	110 (61%)	0.7

^1^n (%)

^2^Pearson's Chi-squared test

Renal and urinary disorders were more likely in males in most of the treatment modalities apart from Temozolomide only treatment, which had no significant sex difference. Females were more likely to develop endocrine disorders in most treatment modalities except for radiation only which did not show any significant sex difference. No significant sex difference in any of the treatment modality observed for the: eye disorders; neoplasms benign, malignant and unspecified including polyps; nervous system disorders, and vascular disorders.

### Adverse events– no treatment

**Fig. 3:**
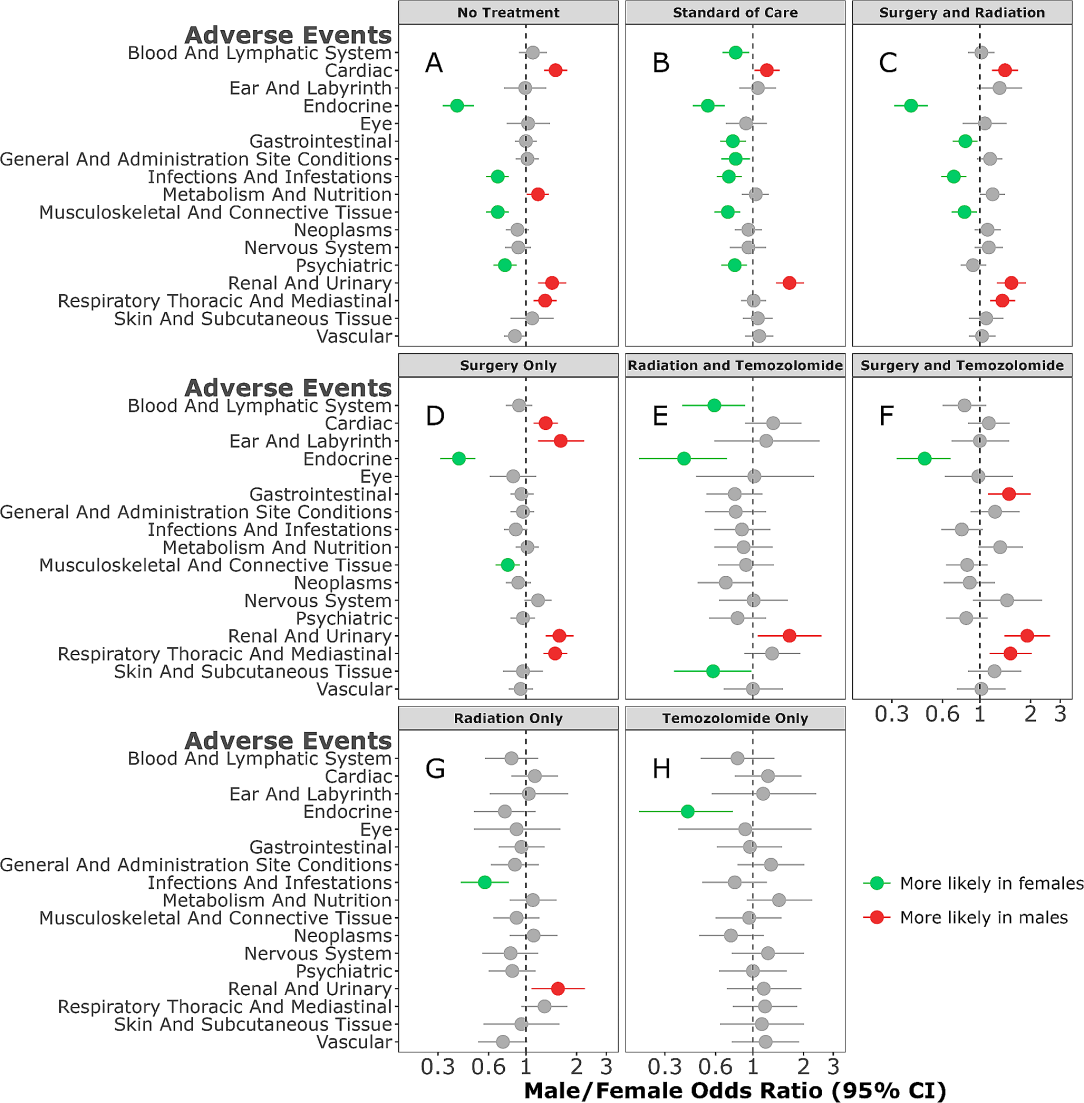
Male/female odds ratio of adverse events for individuals with glioblastoma (GB) receiving no treatment, standard of care (SOC), and other treatment modalities

In patients receiving no treatment, males were more likely to develop cardiac disorders when receiving no treatment (M/F OR = 1.50; 95% CI,1.28–1.76, *p* = < 0.001) and were more likely to develop metabolism and nutrition disorders (M/F OR = 1.18; 95% CI,1.01–1.37, *p* = 0.033) (Fig. 3A). In addition, respiratory, thoracic and mediastinal disorders were more likely in males receiving no treatment (M/F OR = 1.30; 95% CI,1.11–1.52, *p* < 0.001) (Fig. 3A). In contrast, females were more likely to develop musculoskeletal and connective tissue disorders (M/F OR = 0.68; 95% CI,0.58–0.79, *p* < 0.001), infections and infestations (M/F OR = 0.68; 95% CI,0.58–0.79, *p* < 0.001), and psychiatric disorders (M/F OR = 0.75; 95% CI,0.64–0.88, *p* < 0.001) when receiving no treatment (Fig. 3A).

### Adverse events- standard of care

In patients that received SOC, the combination of surgery, followed by concurrent radio-chemotherapy with Temozolomide, females had more types of AE when compared to males (Fig. 3B**).** Males were more likely to develop cardiac disorders (M/F OR = 1.21; 95% CI,1.02–1.44, *p* = 0.029) and renal disorders (M/F OR = 1.65; 95% CI,1.37–2.01, *p* < 0.001) when receiving SOC. Conversely, females were more likely to develop gastrointestinal disorders (M/F OR = 0.76; 95% CI,0.64–0.91, *p* = 0.002) (Fig. 3B) or blood and lymphatic system disorders (M/F OR = 0.79; 95% CI,0.66–0.95, *p* = 0.012) (Fig. 3B**).** Additionally, females were more likely to develop general and administration site conditions (M/F OR = 0.79; 95% CI,0.65–0.96, *p* = 0.02), infections and infestations (M/F OR = 0.72; 95% CI, 0.61–0.86, *p* < 0.001), and psychiatric disorders (M/F OR = 0.78; 95% CI,0.65–0.92, *p* = 0.004) when receiving SOC (Fig. 3B**).**

### Adverse events– other modalities

Most GB patients received other treatment modalities, besides no treatment or SOC, including surgery and radiation, surgery and Temozolomide, radiation and Temozolomide, surgery alone, radiation alone, or Temozolomide only. Sex-differences in AE were also observed in these treatment modalities.

Males were more likely to develop cardiac disorders when receiving the combination of surgery and radiation (Fig. 3C) (M/F OR = 1.41; 95% CI,1.18–1.69, *p* < 0.001) and surgery alone (M/F OR = 1.31; 95% CI,1.11–1.55, *p* = 0.001) (Fig. 3D). Males were also more likely to develop ear and labyrinth disorders when receiving surgery only (M/F OR = 1.61; 95% CI,1.18–2.22, *p* = 0.003) (Fig. 3D) and respiratory, thoracic and mediastinal disorders when receiving the combination of surgery and radiation (M/F OR = 1.36; 95% CI,1.15–1.62, *p* < 0.001) (Fig. 3D), surgery and Temozolomide (M/F OR = 1.52; 95% CI,1.14–2.03, *p* = 0.005) (Fig. 3F) or surgery only (M/F OR = 1.49; 95% CI,1.27–1.76, *p* < 0.001) (Fig. 3D).

Females were more likely to develop gastrointestinal disorders (M/F OR = 0.82; 95% CI,0.69–0.97, *p* = 0.019) or infections and infestations (M/F OR = 0.7; 95% CI,0.59–0.83, *p* < 0.001) when receiving the combination of surgery and radiation (Fig. 3C). In contrast, males were more likely to develop gastrointestinal disorders when having a combination of surgery and Temozolomide (M/F OR = 1.49; 95% CI,1.12-2.00, *p* = 0.007) (Fig. 3F).

In addition, females were more likely to develop blood and lymphatic system disorders (M/F OR = 0.59; 95% CI,0.38–0.9 *p* = 0.016) and skin and subcutaneous tissue disorders (M/F OR = 0.58; 95% CI,0.34–0.98, *p* = 0.042) with a combination of radiation and Temozolomide (Fig. 3E). Musculoskeletal and connective tissue disorders were more likely in females receiving the combination of surgery and radiation (M/F OR = 0.81; 95% CI,0.68–0.96, *p* = 0.016) (Fig. 3C) or surgery only (M/F OR = 0.78; 95% CI,0.66–0.92, *p* = 0.003) (Fig. 3D). In those receiving radiation treatment only, females were more likely to develop infections and infestations (M/F OR = 0.57; 95% CI,0.41–0.79, *p* < 0.001) (Fig. 3G).

## Discussion

Using SEER-Medicare data, this study provides evidence of sex differences in treatment patterns and subsequent AE following a GB diagnosis for adults ages 66 and older in the US. While previous studies have identified sex differences in cancer treatment-induced AEs [26–28], the analyses were limited to single treatment modalities. Here we assessed sex differences in AEs associated with both multimodal and single modality treatment for GB.

Most individuals with GB received either single or bimodal therapy (Table 1). Significantly more males with GB received SOC. In contrast, more females did not receive any type of treatment. This observation has been previously reported in other studies [29, 30]. A difference between those studies and the one here is that we focus on elderly individuals diagnosed with GB rather than patients across the age continuum. This study found that individuals with GB who did not receive treatment were significantly older than those receiving SOC. Further, females with GB who did not receive treatment were significantly older than males receiving no treatment and were more likely to have a higher number of comorbidities (Table 1). These results may suggest that there may be an age bias in the use of SOC to treat GB and that the presence of multiple comorbidities drives treatment decisions.

Elderly individuals often have comorbidities and due to perceived fragility may not be treated in the same way as younger individuals [31, 32]. These combined factors may result in both caregiver and patients being discouraged to pursue aggressive treatments [33]. We were interested to assess whether the differences in age and number of comorbidities in this dataset contributed to the apparent disparity in treatment between males and females with GB who did not receive treatment (Table 1). Further analysis of AE stratified by age group (66–75,75–85, >=85), demonstrated no significant differences between age groups (data not shown).

The Stupp protocol was established as the SOC for GB almost two decades ago [34, 35]. The studies performed establishing GB SOC were performed primarily in GB patients under 70 years of age [4]. Additional studies demonstrated GB SOC benefits in adults 60 years and older [36, 37]. Laperriere and colleagues expanded on these studies suggesting that the same SOC is recommended for individuals 65 and older with a ECOG performance status of 0–2, and suggesting that those older than 65 with higher ECOG scores should consider other treatment approaches [38]. The age and comorbidity scores of the Medicare population may contribute to the low level of individuals receiving SOC in this study.

When observing AEs post GB diagnosis among individuals who did not receive treatment, there are different sex biases observed compared to those who received SOC. While the odds of blood and lymphatic system disorders, gastrointestinal disorders, general and administrative site conditions were higher in females among those who received SOC, there was no statistically significant difference observed by sex among those who did not receive treatment. When looked at together, these findings may suggest that females are more likely to experience a higher variety of AEs after SOC. While these data demonstrate the presence of the sex-differences in AE. It is important to remain cognizant of the fact that these sex differences may be due to, or influenced by, biological, environmental exposure, and behavioral mechanisms.

Previous studies have shown that females receiving Temozolomide have increased risk of experiencing hematological toxicity [39]. In this study, females had significantly higher odds of experiencing blood and lymphatic system disorders when receiving the combination of radiation and Temozolomide. No significant sex differences were observed in other treatment modalities involving the use of Temozolomide. This result may suggest that in the absence of surgery, radiation treatment exacerbates Temozolomide’s potential for blood and lymphatic disorders in females. Deeper analysis into mechanism of such interactions warranted, though is beyond the scope of the current study.

A recent study utilizing mice models suggests that there are glioma-related gut microbiome alterations [40]. Additional studies have demonstrated that Temozolomide treatment further impacts the gut microbiome and that this effect may mitigate the changes induced by brain tumors, possibly through the mitigation of the tumor effects [41]. Based on these preliminary, biological studies, we may expect to see an impact of Temozolomide on gastrointestinal disorder AEs. It is important to note that these studies, did not account for sex as a biological variable. In this study, there were no sex differences in patients who did not receive treatment suggesting that the tumor itself does not promote the sex difference in gastrointestinal disorders observed here.

Further, we found that females had significantly higher odds of experiencing gastrointestinal disorders when receiving the combination of surgery and radiation. However, when given a combination of surgery and Temozolomide, the odds of GI AEs were significantly higher in males. These results may suggest that there may be a sex bias in the development of GI AE. Additional studies adjusting for sex as a biological variable in biological studies, will be required to provide insights on the impact of treatments on the gut microbiome and gastrointestinal related AE.

General and administration site conditions include symptomatic outcomes such as pain or fatigue. Females have been reported to suffer more from chronic pain when compared to men [42]. In addition, studies have demonstrated that female glioma patients suffer from fatigue to a greater extent than males [43]. In our study, there was no sex difference observed in patients who did not receive treatment, suggesting that the presence of the tumor alone did not contribute to a sex based difference in these AE. However, females were more likely to experience general and administration site conditions only after receiving SOC, with no other treatment modality showing a significant sex bias. The general and administration site conditions category consists of categories other than pain and fatigue, so these two AE likely do not contribute entirely to the results observed here. Currently, there is lack of studies focused on general and administration site conditions as a whole category and further analysis is required to dissect the contributing factors for these observations.

Receiving both chemotherapy and radiation therapy has been correlated to hearing problems [44, 45]. Further, surgical procedures in the brain can result in fluid buildup and swelling which may impact ear and labyrinth disorders [46, 47]. Thus, it may not be surprising to find these types of AEs in patients with brain tumors. There were no significant sex differences observed in those individuals receiving no treatment, perhaps suggesting that any ear and labyrinth disorders caused by the tumor itself does not have a sex bias. Interestingly, ear and labyrinth disorders were significantly more likely to occur in males who received surgery only. This may suggest that adverse events to surgery, may have a sex-bias and further studies will be necessary to fully evaluate this potential. While occupational exposures, through employment and lifestyle, may impact sex differences in hearing loss in males, to a higher extent than females, studies have suggest that this difference between males and females becomes nominal at older ages [48]. Studies have also implicated sex-hormone signaling pathways in hearing loss [49, 50]. Thus, it may be also interesting to hypothesize that these mechanisms are involved in the sex-differences in AE to treatment response that we see here, though the exact mechanisms remain to be elucidated.

There are several important limitations to this study. First, this study was limited to claims data associated with Medicare insurance, and it was not possible to identify procedures covered by private insurance, which may create bias. Second, this study assessed only Temozolomide, as it is the most recommended chemotherapy treatment for GB, although there might be an impact from other chemotherapy treatments as well. Third, it was not possible to ensure that surgery and radiation information extracted from the SEER was exactly within 6 months after the diagnosis, as SEER only provides year and month information of the first cancer therapy started without further time information on subsequent therapies. Next, the nature of Medicare beneficiaries prevents the collection of data prior to Medicare enrollment, which potentially limits the available amount of comorbidity data beyond one year prior to diagnosis. Finally, as GB can occur at any age, these results are not generalizable across all ages and are instead informative for older US adults only.

## Conclusions

In conclusion, our study found that sex differences exist in GB treatment receipt. Males with GB were more likely to receive SOC. Across different treatment modalities sex differences exist in the odds of experiencing an AE for individuals 66 and older diagnosed with GB. Females were more likely to develop more types of AEs after SOC compared to males. While sex differences exist in occupational/environmental exposures, social behavior, seeking medical care, and types of comorbidities, and are important aspects to analyze, this work is not intended to begin to elucidate how these pre-treatment differences may directly impact treatment response. Nonetheless, regardless of the underlying mechanism, this work demonstrates that there are sex differences in AE in response to treatment. Thus, health care providers should consider how sex biases may impact treatment as well as the potential of developing an AE when discussing and developing treatment plans for individuals 66 or older with GB.

### Electronic supplementary material

Below is the link to the electronic supplementary material.

**Supplementary Material 1**: **Supplemental Figure 1**: Correlation Matrix Between Each AE Used in This Study. Correlation between each adverse event developed by individuals with glioblastoma is calculated and shown in a matrix format. More intense red color indicates higher correlation between the adverse events

**Supplementary Material 2**: **Supplementary Table 1**. Codes Used to Identify Treatment Patterns. Table contains treatment codes that were used to identify 3 treatment categories: surgery, radiation and Temozolomide. **Supplementary Table 2**. CTCAE AE categories. Adverse events with corresponding medDRA codes that were used in this study

## Data Availability

The datasets used to conduct this study are available upon approval of a research protocol from the National Cancer Institute SEER-Medicare Program. Instructions for obtaining these data are available at https://healthcaredelivery.cancer.gov/seermedicare/obtain/.
