# Supplementary material for: Sex differences in adverse events in Medicare individuals ≥ 66 years of age post glioblastoma treatment
Source: J Neurooncol. 2024 Apr 2;168(1):111–23. doi: 10.1007/s11060-024-04652-z (PMC11093825; doi:10.1007/s11060-024-04652-z)

**Supplemental Figure 1:** Correlation Matrix Between Each AE Used in This Study.

Correlation between each adverse event developed by individuals with glioblastoma is calculated and shown in a matrix format. More intense red color indicates higher correlation between the adverse events.


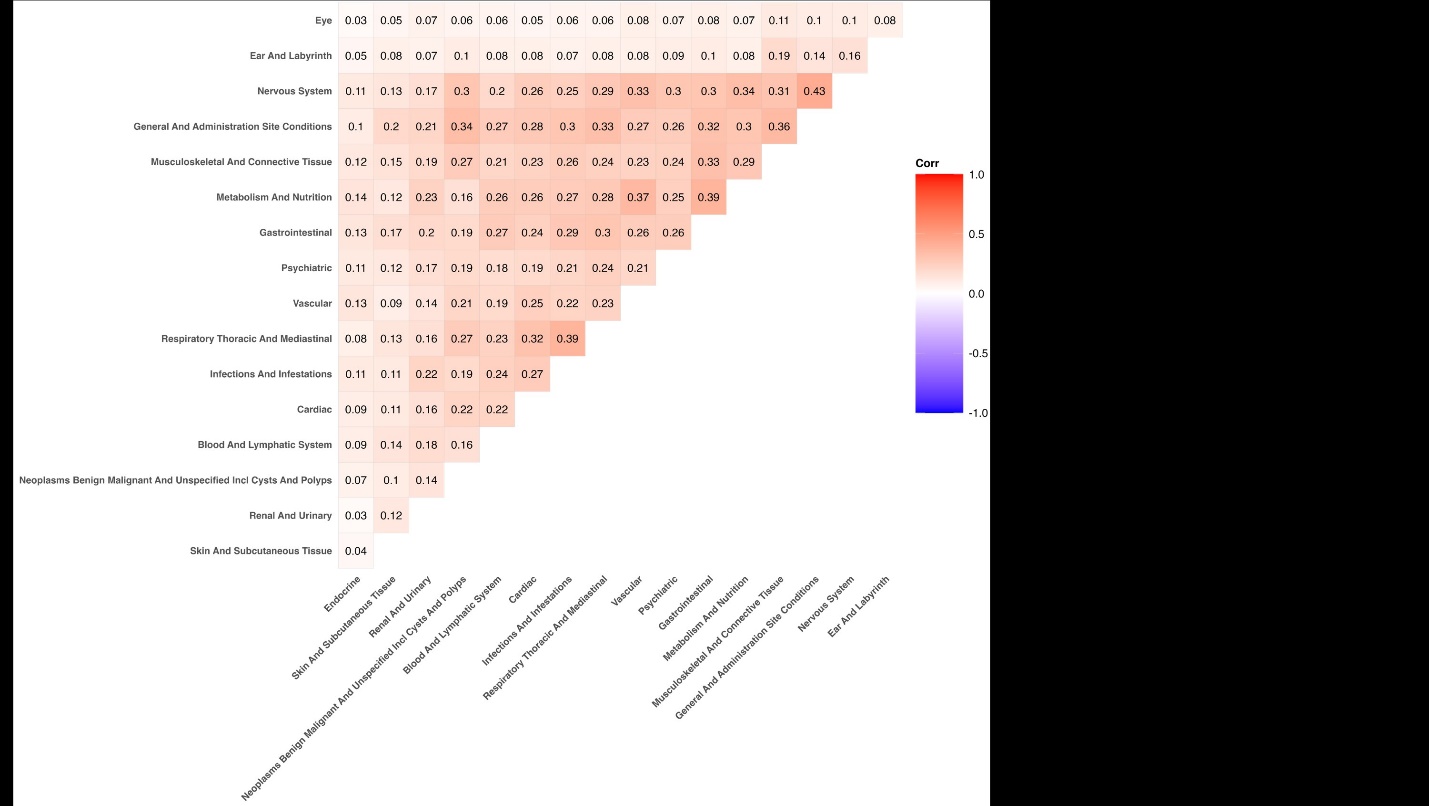

Supplement: Supplementary file 1 — Supplementary Material 1: Supplemental Figure 1: Correlation Matrix Between Each AE Used in This Study. Correlation between each adverse event developed by individuals with glioblastoma is calculated and shown in a matrix format. More intense red color indicates higher correlation between the adverse events [file 11060_2024_4652_MOESM1_ESM.docx]
