# Supplementary material for: Sex differences in adverse events in Medicare individuals ≥ 66 years of age post glioblastoma treatment
Source: J Neurooncol. 2024 Apr 2;168(1):111–23. doi: 10.1007/s11060-024-04652-z (PMC11093825; doi:10.1007/s11060-024-04652-z)
Supplement: Supplementary file 2 — Supplementary Material 2: Supplementary Table 1. Codes Used to Identify Treatment Patterns. Table contains treatment codes that were used to identify 3 treatment categories: surgery, radiation and Temozolomide. Supplementary Table 2. CTCAE AE categories. Adverse events with corresponding medDRA codes that were used in this study [file 11060_2024_4652_MOESM2_ESM.docx]

Supplementary Table 1. Codes Used to Identify Treatment Patterns.

Table contains treatment codes that were used to identify 3 treatment categories: surgery, radiation and Temozolomide.

| 9381, 9400, 9410, 9411, 9420, 9442, 9401, 9440, 9441, 9442, 9445, 9450, 9451, 9460, 9382, 9421, 9425, 9384, 9424, 9431, 9383, 9391, 9392- 9394, 9396, 9380, 9385, 9423, 9430, 9444, 8680, 8681, 8690, 8693, 9412, 9413, 9442, 9490, 9492, 9493, 9505, 9506, 9509, 9522, 9523, 9390, 9360, 9361, 9362, 9395, 8963, 9364, 9470-9478, 9480, 9500, 9501, 9502, 9508, 9540, 9541, 9550, 9560, 9561, 9570, 9571, 9562, 9563, 9530-9535, 9537- 9539, 8324, 8710, 8711, 8800-8806, 8810, 8811, 8815, 8821, 8824, 8825, 8830, 8831, 8835, 8836, 8840, 8850-8854, 8857, 8861, 8870, 8880, 8890, 8897, 8900-8902, 8910, 8912, 8920, 8921, 8935, 8990, 9040, 9120, 9125, 9130, 9131, 9133, 9136, 9150, 9161, 9170, 9180, 9210, 9220, 9231, 9240, 9241, 9243, 9260, 9370-9373, 8720, 8728, 8770, 9590, 9591, 9596, 9650-9655, 9659, 9661-9665, 9667, 9670, 9671, 9673, 9675, 9680, 9684, 9687, 9688, 9690, 9691, 9695, 9698, 9699, 9701, 9702, 9705, 9712, 9714, 9715, 9719, 9724, 9727-9729, 9735, 9737, 9738, 9750, 9751, 9755, 9756, 9811-9819, 9823, 9826, 9827, 9831, 9832, 9837, 9861, 9866, 9930, 9965, 9966, 9967, 9970, 9971, 9975, 9731, 9733, 9734, 9740, 9741, 9749, 9752-9754, 9757-9758, 9759, 9760, 9766, 9860, 8440, 9060, 9061, 9064, 9065, 9070-9072, 9080-9083, 9084, 9085, 9100, 9101, 8040, 8140, 8146, 8246, 8260, 8270-8272, 8280, 8281, 8290, 8300, 8310, 8323, 9391, 9432 , 9492, 9580, 9582, 9350-9352, 9121-9123, 9133, 9140, 8000-8005, 8010, 8020, 8021, 8320, 8452, 8713, 8896, 8963, 8980, 9084, 9173, 9363, 9503 |
| --- |

**Supplementary Table 2.** CTCAE AE categories. Adverse events with corresponding medDRA codes that were used in this study.

| **Treatment Type** | **Code Classification** | **Code** |
| --- | --- | --- |
| Radiation Therapy | ICD-9-CM | 92.23–92.24, 92.30–92.33, and 92.39. |
|  | ICD10 | D01097Z, D01098Z, D01099Z, D0109BZ, D0109CZ, D0109YZ, D010B7Z, D010B8Z, D010B9Z, D010BBZ, D010BCZ, D010BYZ, D01197Z, D01198Z, D01199Z, D0119BZ, D0119CZ, D0119YZ, D011B7Z, D011B8Z, D011B9Z, D011BBZ, D011BCZ, D011BYZ, D01697Z, D01698Z, D01699Z, D0169BZ, D0169CZ, D0169YZ, D016B7Z, D016B8Z, D016B9Z, D016BBZ, D016BCZ, D016BYZ, D01797Z, D01798Z, D01799Z, D0179BZ, D0179CZ, D0179YZ, D017B7Z, D017B8Z, D017B9Z, D017BBZ, D017BCZ, D017BYZ, D71097Z, D71098Z, D71099Z, D7109BZ, D7109CZ, D7109YZ, D710B7Z, D710B8Z, D710B9Z, D710BBZ, D710BCZ, D710BYZ, D71197Z, D71198Z, D71199Z, D7119BZ, D7119CZ, D7119YZ, D711B7Z, D711B8Z, D711B9Z, D711BBZ, D711BCZ, D711BYZ, D71297Z, D71298Z, D71299Z, D7129BZ, D7129CZ, D7129YZ, D712B7Z, D712B8Z, D712B9Z, D712BBZ, D712BCZ, D712BYZ, D71397Z, D71398Z, D71399Z, D7139BZ, D7139CZ, D7139YZ, D713B7Z, D713B8Z, D713B9Z, D713BBZ, D713BCZ, D713BYZ, D71497Z, D71498Z, D71499Z, D7149BZ, D7149CZ, D7149YZ, D714B7Z, D714B8Z, D714B9Z, D714BBZ, D714BCZ, D714BYZ, D71597Z, D71598Z, D71599Z, D7159BZ, D7159CZ, D7159YZ, D715B7Z, D715B8Z, D715B9Z, D715BBZ, D715BCZ, D715BYZ, D71697Z, D71698Z, D71699Z, D7169BZ, D7169CZ, D7169YZ, D716B7Z, D716B8Z, D716B9Z, D716BBZ, D716BCZ, D716BYZ, D71797Z, D71798Z, D71799Z, D7179BZ, D7179CZ, D7179YZ, D717B7Z, D717B8Z, D717B9Z, D717BBZ, D717BCZ, D717BYZ, D71897Z, D71898Z, D71899Z, D7189BZ, D7189CZ, D7189YZ, D718B7Z, D718B8Z, D718B9Z, D718BBZ, D718BCZ, D718BYZ, D81097Z, D81098Z, D81099Z, D8109BZ, D8109CZ, D8109YZ, D810B7Z, D810B8Z, D810B9Z, D810BBZ, D810BCZ, D810BYZ, D91097Z, D91098Z, D91099Z, D9109BZ, D9109CZ, D9109YZ, D910B7Z, D910B8Z, D910B9Z, D910BBZ, D910BCZ, D910BYZ, D91197Z, D91198Z, D91199Z, D9119BZ, D9119CZ, D9119YZ, D911B7Z, D911B8Z, D911B9Z, D911BBZ, D911BCZ, D911BYZ, D91397Z, D91398Z, D91399Z, D9139BZ, D9139CZ, D9139YZ, D913B7Z, D913B8Z, D913B9Z, D913BBZ, D913BCZ, D913BYZ, D91497Z, D91498Z, D91499Z, D9149BZ, D9149CZ, D9149YZ, D914B7Z, D914B8Z, D914B9Z, D914BBZ, D914BCZ, D914BYZ, D91597Z, D91598Z, D91599Z, D9159BZ, D9159CZ, D9159YZ, D915B7Z, D915B8Z, D915B9Z, D915BBZ, D915BCZ, D915BYZ, D91697Z, D91698Z, D91699Z, D9169BZ, D9169CZ, D9169YZ, D916B7Z, D916B8Z, D916B9Z, D916BBZ, D916BCZ, D916BYZ, D91797Z, D91798Z, D91799Z, D9179BZ, D9179CZ, D9179YZ, D917B7Z, D917B8Z, D917B9Z, D917BBZ, D917BCZ, D917BYZ, D91897Z, D91898Z, D91899Z, D9189BZ, D9189CZ, D9189YZ, D918B7Z, D918B8Z, D918B9Z, D918BBZ, D918BCZ, D918BYZ, D91997Z, D91998Z, D91999Z, D9199BZ, D9199CZ, D9199YZ, D919B7Z, D919B8Z, D919B9Z, D919BBZ, D919BCZ, D919BYZ, D91B97Z, D91B98Z, D91B99Z, D91B9BZ, D91B9CZ, D91B9YZ, D91BB7Z, D91BB8Z, D91BB9Z, D91BBBZ, D91BBCZ, D91BBYZ, D91D97Z, D91D98Z, D91D99Z, D91D9BZ, D91D9CZ, D91D9YZ, D91DB7Z, D91DB8Z, D91DB9Z, D91DBBZ, D91DBCZ, D91DBYZ, D91F97Z, D91F98Z, D91F99Z, D91F9BZ, D91F9CZ, D91F9YZ, D91FB7Z, D91FB8Z, D91FB9Z, D91FBBZ, D91FBCZ, D91FBYZ, DB1097Z, DB1098Z, DB1099Z, DB109BZ, DB109CZ, DB109YZ, DB10B7Z, DB10B8Z, DB10B9Z, DB10BBZ, DB10BCZ, DB10BYZ, DB1197Z, DB1198Z, DB1199Z, DB119BZ, DB119CZ, DB119YZ, DB11B7Z, DB11B8Z, DB11B9Z, DB11BBZ, DB11BCZ, DB11BYZ, DB1297Z, DB1298Z, DB1299Z, DB129BZ, DB129CZ, DB129YZ, DB12B7Z, DB12B8Z, DB12B9Z, DB12BBZ, DB12BCZ, DB12BYZ, DB1597Z, DB1598Z, DB1599Z, DB159BZ, DB159CZ, DB159YZ, DB15B7Z, DB15B8Z, DB15B9Z, DB15BBZ, DB15BCZ, DB15BYZ, DB1697Z, DB1698Z, DB1699Z, DB169BZ, DB169CZ, DB169YZ, DB16B7Z, DB16B8Z, DB16B9Z, DB16BBZ, DB16BCZ, DB16BYZ, DB1797Z, DB1798Z, DB1799Z, DB179BZ, DB179CZ, DB179YZ, DB17B7Z, DB17B8Z, DB17B9Z, DB17BBZ, DB17BCZ, DB17BYZ, DB1897Z, DB1898Z, DB1899Z, DB189BZ, DB189CZ, DB189YZ, DB18B7Z, DB18B8Z, DB18B9Z, DB18BBZ, DB18BCZ, DB18BYZ, DD1097Z, DD1098Z, DD1099Z, DD109BZ, DD109CZ, DD109YZ, DD10B7Z, DD10B8Z, DD10B9Z, DD10BBZ, DD10BCZ, DD10BYZ, DD1197Z, DD1198Z, DD1199Z, DD119BZ, DD119CZ, DD119YZ, DD11B7Z, DD11B8Z, DD11B9Z, DD11BBZ, DD11BCZ, DD11BYZ, DD1297Z, DD1298Z, DD1299Z, DD129BZ, DD129CZ, DD129YZ, DD12B7Z, DD12B8Z, DD12B9Z, DD12BBZ, DD12BCZ, DD12BYZ, DD1397Z, DD1398Z, DD1399Z, DD139BZ, DD139CZ, DD139YZ, DD13B7Z, DD13B8Z, DD13B9Z, DD13BBZ, DD13BCZ, DD13BYZ, DD1497Z, DD1498Z, DD1499Z, DD149BZ, DD149CZ, DD149YZ, DD14B7Z, DD14B8Z, DD14B9Z, DD14BBZ, DD14BCZ, DD14BYZ, DD1597Z, DD1598Z, DD1599Z, DD159BZ, DD159CZ, DD159YZ, DD15B7Z, DD15B8Z, DD15B9Z, DD15BBZ, DD15BCZ, DD15BYZ, DD1797Z, DD1798Z, DD1799Z, DD179BZ, DD179CZ, DD179YZ, DD17B7Z, DD17B8Z, DD17B9Z, DD17BBZ, DD17BCZ, DD17BYZ, DF1097Z, DF1098Z, DF1099Z, DF109BZ, DF109CZ, DF109YZ, DF10B7Z, DF10B8Z, DF10B9Z, DF10BBZ, DF10BCZ, DF10BYZ, DF1197Z, DF1198Z, DF1199Z, DF119BZ, DF119CZ, DF119YZ, DF11B7Z, DF11B8Z, DF11B9Z, DF11BBZ, DF11BCZ, DF11BYZ, DF1297Z, DF1298Z, DF1299Z, DF129BZ, DF129CZ, DF129YZ, DF12B7Z, DF12B8Z, DF12B9Z, DF12BBZ, DF12BCZ, DF12BYZ, DF1397Z, DF1398Z, DF1399Z, DF139BZ, DF139CZ, DF139YZ, DF13B7Z, DF13B8Z, DF13B9Z, DF13BBZ, DF13BCZ, DF13BYZ, DG1097Z, DG1098Z, DG1099Z, DG109BZ, DG109CZ, DG109YZ, DG10B7Z, DG10B8Z, DG10B9Z, DG10BBZ, DG10BCZ, DG10BYZ, DG1197Z, DG1198Z, DG1199Z, DG119BZ, DG119CZ, DG119YZ, DG11B7Z, DG11B8Z, DG11B9Z, DG11BBZ, DG11BCZ, DG11BYZ, DG1297Z, DG1298Z, DG1299Z, DG129BZ, DG129CZ, DG129YZ, DG12B7Z, DG12B8Z, DG12B9Z, DG12BBZ, DG12BCZ, DG12BYZ, DG1497Z, DG1498Z, DG1499Z, DG149BZ, DG149CZ, DG149YZ, DG14B7Z, DG14B8Z, DG14B9Z, DG14BBZ, DG14BCZ, DG14BYZ, DG1597Z, DG1598Z, DG1599Z, DG159BZ, DG159CZ, DG159YZ, DG15B7Z, DG15B8Z, DG15B9Z, DG15BBZ, DG15BCZ, DG15BYZ, DM1097Z, DM1098Z, DM1099Z, DM109BZ, DM109CZ, DM109YZ, DM10B7Z, DM10B8Z, DM10B9Z, DM10BBZ, DM10BCZ, DM10BYZ, DM1197Z, DM1198Z, DM1199Z, DM119BZ, DM119CZ, DM119YZ, DM11B7Z, DM11B8Z, DM11B9Z, DM11BBZ, DM11BCZ, DM11BYZ, DT1097Z, DT1098Z, DT1099Z, DT109BZ, DT109CZ, DT109YZ, DT10B7Z, DT10B8Z, DT10B9Z, DT10BBZ, DT10BCZ, DT10BYZ, DT1197Z, DT1198Z, DT1199Z, DT119BZ, DT119CZ, DT119YZ, DT11B7Z, DT11B8Z, DT11B9Z, DT11BBZ, DT11BCZ, DT11BYZ, DT1297Z, DT1298Z, DT1299Z, DT129BZ, DT129CZ, DT129YZ, DT12B7Z, DT12B8Z, DT12B9Z, DT12BBZ, DT12BCZ, DT12BYZ, DT1397Z, DT1398Z, DT1399Z, DT139BZ, DT139CZ, DT139YZ, DT13B7Z, DT13B8Z, DT13B9Z, DT13BBZ, DT13BCZ, DT13BYZ, DU1097Z, DU1098Z, DU1099Z, DU109BZ, DU109CZ, DU109YZ, DU10B7Z, DU10B8Z, DU10B9Z, DU10BBZ, DU10BCZ, DU10BYZ, DU1197Z, DU1198Z, DU1199Z, DU119BZ, DU119CZ, DU119YZ, DU11B7Z, DU11B8Z, DU11B9Z, DU11BBZ, DU11BCZ, DU11BYZ, DU1297Z, DU1298Z, DU1299Z, DU129BZ, DU129CZ, DU129YZ, DU12B7Z, DU12B8Z, DU12B9Z, DU12BBZ, DU12BCZ, DU12BYZ, DV1097Z, DV1098Z, DV1099Z, DV109BZ, DV109CZ, DV109YZ, DV10B7Z, DV10B8Z, DV10B9Z, DV10BBZ, DV10BCZ, DV10BYZ, DV1197Z, DV1198Z, DV1199Z, DV119BZ, DV119CZ, DV119YZ, DV11B7Z, DV11B8Z, DV11B9Z, DV11BBZ, DV11BCZ, DV11BYZ, DW1197Z, DW1198Z, DW1199Z, DW119BZ, DW119CZ, DW119YZ, DW11B7Z, DW11B8Z, DW11B9Z, DW11BBZ, DW11BCZ, DW11BYZ, DW1297Z, DW1298Z, DW1299Z, DW129BZ, DW129CZ, DW129YZ, DW12B7Z, DW12B8Z, DW12B9Z, DW12BBZ, DW12BCZ, DW12BYZ, DW1397Z, DW1398Z, DW1399Z, DW139BZ, DW139CZ, DW139YZ, DW13B7Z, DW13B8Z, DW13B9Z, DW13BBZ, DW13BCZ, DW13BYZ, DW1697Z, DW1698Z, DW1699Z, DW169BZ, DW169CZ, DW169YZ, DW16B7Z, DW16B8Z, DW16B9Z, DW16BBZ, DW16BCZ, DW16BYZ, D0000ZZ, D0001ZZ, D0002ZZ, D0010ZZ, D0011ZZ, D0012ZZ, D0060ZZ, D0061ZZ, D0062ZZ, D0070ZZ, D0071ZZ, D0072ZZ, D7000ZZ, D7001ZZ, D7002ZZ, D7010ZZ, D7011ZZ, D7012ZZ, D7020ZZ, D7021ZZ, D7022ZZ, D7030ZZ, D7031ZZ, D7032ZZ, D7040ZZ, D7041ZZ, D7042ZZ, D7050ZZ, D7051ZZ, D7052ZZ, D7060ZZ, D7061ZZ, D7062ZZ, D7070ZZ, D7071ZZ, D7072ZZ, D7080ZZ, D7081ZZ, D7082ZZ, D8000ZZ, D8001ZZ, D8002ZZ, D9000ZZ, D9001ZZ, D9002ZZ, D9010ZZ, D9011ZZ, D9012ZZ, D9030ZZ, D9031ZZ, D9032ZZ, D9040ZZ, D9041ZZ, D9042ZZ, D9050ZZ, D9051ZZ, D9052ZZ, D9060ZZ, D9061ZZ, D9062ZZ, D9070ZZ, D9071ZZ, D9072ZZ, D9080ZZ, D9081ZZ, D9082ZZ, D9090ZZ, D9091ZZ, D9092ZZ, D90B0ZZ, D90B1ZZ, D90B2ZZ, D90D0ZZ, D90D1ZZ, D90D2ZZ, D90F0ZZ, D90F1ZZ, D90F2ZZ, DB000ZZ, DB001ZZ, DB002ZZ, DB010ZZ, DB011ZZ, DB012ZZ, DB020ZZ, DB021ZZ, DB022ZZ, DB050ZZ, DB051ZZ, DB052ZZ, DB060ZZ, DB061ZZ, DB062ZZ, DB070ZZ, DB071ZZ, DB072ZZ, DB080ZZ, DB081ZZ, DB082ZZ, DD000ZZ, DD001ZZ, DD002ZZ, DD010ZZ, DD011ZZ, DD012ZZ, DD020ZZ, DD021ZZ, DD022ZZ, DD030ZZ, DD031ZZ, DD032ZZ, DD040ZZ, DD041ZZ, DD042ZZ, DD050ZZ, DD051ZZ, DD052ZZ, DD070ZZ, DD071ZZ, DD072ZZ, DF000ZZ, DF001ZZ, DF002ZZ, DF010ZZ, DF011ZZ, DF012ZZ, DF020ZZ, DF021ZZ, DF022ZZ, DF030ZZ, DF031ZZ, DF032ZZ, DG000ZZ, DG001ZZ, DG002ZZ, DG010ZZ, DG011ZZ, DG012ZZ, DG020ZZ, DG021ZZ, DG022ZZ, DG040ZZ, DG041ZZ, DG042ZZ, DG050ZZ, DG051ZZ, DG052ZZ, DH020ZZ, DH021ZZ, DH022ZZ, DH030ZZ, DH031ZZ, DH032ZZ, DH040ZZ, DH041ZZ, DH042ZZ, DH060ZZ, DH061ZZ, DH062ZZ, DH070ZZ, DH071ZZ, DH072ZZ, DH080ZZ, DH081ZZ, DH082ZZ, DH090ZZ, DH091ZZ, DH092ZZ, DH0B0ZZ, DH0B1ZZ, DH0B2ZZ, DM000ZZ, DM001ZZ, DM002ZZ, DM010ZZ, DM011ZZ, DM012ZZ, DP000ZZ, DP001ZZ, DP002ZZ, DP020ZZ, DP021ZZ, DP022ZZ, DP030ZZ, DP031ZZ, DP032ZZ, DP040ZZ, DP041ZZ, DP042ZZ, DP050ZZ, DP051ZZ, DP052ZZ, DP060ZZ, DP061ZZ, DP062ZZ, DP070ZZ, DP071ZZ, DP072ZZ, DP080ZZ, DP081ZZ, DP082ZZ, DP090ZZ, DP091ZZ, DP092ZZ, DP0B0ZZ, DP0B1ZZ, DP0B2ZZ, DP0C0ZZ, DP0C1ZZ, DP0C2ZZ, DT000ZZ, DT001ZZ, DT002ZZ, DT010ZZ, DT011ZZ, DT012ZZ, DT020ZZ, DT021ZZ, DT022ZZ, DT030ZZ, DT031ZZ, DT032ZZ, DU000ZZ, DU001ZZ, DU002ZZ, DU010ZZ, DU011ZZ, DU012ZZ, DU020ZZ, DU021ZZ, DU022ZZ, DV000ZZ, DV001ZZ, DV002ZZ, DV010ZZ, DV011ZZ, DV012ZZ, DW010ZZ, DW011ZZ, DW012ZZ, DW020ZZ, DW021ZZ, DW022ZZ, DW030ZZ, DW031ZZ, DW032ZZ, DW040ZZ, DW041ZZ, DW042ZZ, DW050ZZ, DW051ZZ, DW052ZZ, DW060ZZ, DW061ZZ, DW062ZZ, D020DZZ, D021DZZ, D027DZZ, DG20DZZ, D020DZZ, D021DZZ, D027DZZ, DG20DZZ, D020JZZ, D021JZZ, D027JZZ, DG20JZZ, D020HZZ, D021HZZ, D027HZZ, DG20HZZ, D020DZZ, D021DZZ, D027DZZ, DG20DZZ |
|  | CPT | 77261-77263, 77280, 77285, 77290, 77295, 77299–77301, 77305, 77310, 77315, 77321, 77332-77334, 77336-77337, 77370-77372, 77399, 77402-77414, 77416, 77418-77420, 77425, 77427, 77430, 77432, and 0073T. |
|  | HCPCS | G0173-G0174, G0242-G0243, G0251, and G0338-G0340. |
| Neurosurgical resection | ICD-9-CM | 01.21–01.25, 01.31, 01.51, and 01.59. |
|  | ICD10 | 0N9000Z, 0N900ZZ, 0N9030Z, 0N903ZZ, 0N9040Z, 0N904ZZ, 00P00MZ, 00P03MZ, 00P04MZ, 00P60MZ, 00P63MZ, 00P64MZ, 00P6XMZ, 0NP00MZ, 0NP03MZ, 0NP04MZ, 0NP0XMZ, 0WJ10ZZ, 00J00ZZ, 00W00JZ, 00W00KZ, 0N800ZZ, 0N803ZZ, 0N804ZZ, 0NC10ZZ, 0NC13ZZ, 0NC14ZZ, 0NC20ZZ, 0NC23ZZ, 0NC24ZZ, 0NC30ZZ, 0NC33ZZ, 0NC34ZZ, 0NC40ZZ, 0NC43ZZ, 0NC44ZZ, 0NC50ZZ, 0NC53ZZ, 0NC54ZZ, 0NC60ZZ, 0NC63ZZ, 0NC64ZZ, 0NC70ZZ, 0NC73ZZ, 0NC74ZZ, 0NC80ZZ, 0NC83ZZ, 0NC84ZZ, 0NH00MZ, 0NH03MZ, 0NH04MZ, 0NP000Z, 0NP004Z, 0NP005Z, 0NP007Z, 0NP00KZ, 0NP00SZ, 0NP030Z, 0NP034Z, 0NP037Z, 0NP03KZ, 0NP03SZ, 0NP040Z, 0NP044Z, 0NP047Z, 0NP04KZ, 0NP04SZ, 0NP0X4Z, 0NP0XSZ, 0NW000Z, 0NW004Z, 0NW005Z, 0NW007Z, 0NW00JZ, 0NW00KZ, 0NW00MZ, 0NW00SZ, 0NW030Z, 0NW034Z, 0NW035Z, 0NW037Z, 0NW03JZ, 0NW03KZ, 0NW03MZ, 0NW03SZ, 0NW040Z, 0NW044Z, 0NW045Z, 0NW047Z, 0NW04JZ, 0NW04KZ, 0NW04MZ, 0NW04SZ, 0W9100Z, 0W910ZZ, 0WC10ZZ, 0WC13ZZ, 0WC14ZZ, 0WH10YZ, 0WH13YZ, 0WH14YZ, 0WJ10ZZ, 0WP100Z, 0WP101Z, 0WP10JZ, 0WP10YZ, 0WP130Z, 0WP131Z, 0WP13JZ, 0WP13YZ, 0WP140Z, 0WP141Z, 0WP14JZ, 0WP14YZ, 0WW100Z, 0WW101Z, 0WW103Z, 0WW10JZ, 0WW10YZ, 0WW130Z, 0WW131Z, 0WW133Z, 0WW13JZ, 0WW13YZ, 0WW140Z, 0WW141Z, 0WW143Z, 0WW14JZ, 0WW14YZ, 0N500ZZ, 0N503ZZ, 0N504ZZ, 0NB00ZZ, 0NB03ZZ, 0NB04ZZ, 0NT10ZZ, 0NT20ZZ, 0NT30ZZ, 0NT40ZZ, 0NT50ZZ, 0NT60ZZ, 0NT70ZZ, 0NT80ZZ, 009100Z, 00910ZZ, 00C10ZZ, 00C13ZZ, 00C14ZZ, 00510ZZ, 00513ZZ, 00514ZZ, 00B10ZZ, 00B13ZZ, 00B14ZZ, 00D10ZZ, 00D13ZZ, 00D14ZZ, 00500ZZ, 00503ZZ, 00504ZZ, 00B00ZZ, 00B03ZZ, 00B04ZZ |
|  | CPT | 61304–61305, 61312–61315, 61320–61321, 61330, 61332–61334, 61340, 61343, 61345, 61440, 61450, 61458, 61460, 61470, 61500–61501, 61510, 61512, 61514, 61516, 61518, 61519- 61522, 61524, 61526, 61530–61531, 61533–61536, 61538-61539, 61541–61546, 61550, 61552, 61556–61559, 61563- 61564, 61570–61571, 61575–61576, 61580–61586, 61590–61592, 61596–61598, 61600–61601, 61605–61613, and 61615–61616. |
| **Chemotherapies** |  |  |
| Temozolomide | HCPCS | C1086, C9253, J8700, J9328 |
|  | NDC | 54868-4142, 54868-5348, 54868-5350, 54868-5354, 54868-5980, 62175-0240, 62175-0241, 62175-0242, 62175-0243, 62175-0244, 62175-0245, 64144-0501, 64144-0502, 64144-0503, 64144-0504, 64144-0505, 64144-0506, 64980-0333, 64980-0334, 64980-0335, 64980-0336, 64980-0337, 64980-0338, 65162-0801, 65162-0802, 65162-0803, 65162-0804, 65162-0805, 65162-0806, 67877-0537, 67877-0538, 67877-0539, 67877-0540, 67877-0541, 67877-0542, 69189-7638, 00085-0381, 16729-0048, 16729-0050, 16729-0051, 16729-0129, 16729-0130, 40051-0604, 40051-0605, 40051-0606, 40051-0607, 40051-0608, 40051-0609, 47335-0893, 00054-0320, 00054-0321, 00054-0322, 00054-0323, 00054-0324, 00054-0325, 16729-0049, 50268-0761, 50268-0762, 47335-0890, 47335-0891, 47335-0892, 47335-0929, 47335-0930, 62559-0921, 62559-0920, 62559-0922, 62559-0923, 62559-0924, 62559-0925, 00085-3004, 00085-1366, 00085-1381, 00085-1417, 00085-1425, 00085-1430, 00085-1519, 00093-7599, 00093-7600, 00093-7601, 00093-7602, 00093-7638, 00093-7639, 00378-5260, 00378-5261, 00378-5262, 00378-5263, 00378-5264, 00378-5265, 00527-1777, 00527-1778, 00527-1779, 00527-1780, 00527-1781, 00527-1782, 00781-2691, 00781-2692, 00781-2693, 00781-2694, 00781-2695, 00781-2696, 42737-0101, 42737-0102, 42737-0103, 42737-0104, 42737-0105, 42737-0106, 43975-0252, 43975-0253, 43975-0254, 43975-0255, 43975-0257, 50268-0763, 51862-0083, 51862-0084, 51862-0085, 51862-0086, 51862-0087, 51862-0088, 75834-0132, 75834-0142, 75834-0143, 75834-0144, 75834-0145, 43975-0256, 59923-0703, 59923-0704, 59923-0705, 59923-0706, 59923-0707, 59923-0708, 59923-0709, 59923-0710, 59923-0711, 59923-0712, 59923-0713, |

**Supplemental table 3.**

| **AE** | **MedDRA Code** |
| --- | --- |
| Blood and lymphatic system disorders | 10002272, 10005329, 10048580, 10013442, 10014950, 10016288, 10019491, 10019515, 10024378, 10025182, 10027506, 10043648 |
| Cardiac disorders | 10061589, 10003586, 10003658, 10003662, 10003673, 10003674, 10007515, 10007541, 10008481, 10010276, 10011703, 10019279, 10069501, 10061532, 10027786, 10027787, 10028596, 10028606, 10033557, 10034040, 10034474, 10053565, 10034484, 10061541, 10038748, 10058597, 10040639, 10040741, 10040752, 10042604, 10061389, 10047281, 10047290, 10047302 |
| Ear and labyrinth disorders | 10013993, 10014020, 10065785, 10019245, 10065838, 10043882, 10047340, 10047386 |
| Endocrine disorders | 10001367, 10011655, 10012205, 10014698, 10018746, 10020705, 10020850, 10021041, 10062767, 10021067, 10021114, 10058084, 10067734, 10047488 |
| Eye disorders | 10005886, 10007739, 10048492, 10013774, 10015829, 10015919, 10015958, 10061145, 10016757, 10016778, 10018304, 10023332, 10029404, 10061322, 10033703, 10054541, 10034960, 10038848, 10038897, 10038901, 10038923, 10061510, 10046851, 10047516, 10047656, 10047848 |
| Gastrointestinal disorders | 10000060, 10000081, 10002153, 10002156, 10055226, 10065721, 10065722, 10002167, 10002176, 10002180, 10003445, 10004222, 10005265, 10065747, 10008417, 10009167, 10009887, 10009995, 10009998, 10010000, 10010001, 10010004, 10010006, 10010774, 10012318, 10012727, 10013781, 10013828, 10055242, 10013830, 10013832, 10050094, 10013836, 10013946, 10013950, 10014893, 10062570, 10065851, 10015384, 10065727, 10015387, 10015388, 10055472, 10015448, 10015451, 10015453, 10015461, 10016296, 10016766, 10065713, 10017789, 10051886, 10017815, 10061970, 10017822, 10017853, 10066874, 10017947, 10017877, 10017999, 10018043, 10018286, 10060640, 10019611, 10065728, 10055287, 10065730, 10021305, 10021307, 10021309, 10021328, 10055291, 10065719, 10055300, 10065732, 10023174, 10023176, 10023177, 10024561, 10051746, 10025476, 10028130, 10028813, 10029957, 10065720, 10054520, 10030980, 10031009, 10065703, 10049192, 10033626, 10058096, 10033645, 10034536, 10065704, 10036774, 10064993, 10038062, 10038064, 10063190, 10065709, 10065707, 10038072, 10038073, 10038079, 10038080, 10038981, 10056681, 10039411, 10065710, 10041101, 10041103, 10062263, 10041133, 10042112, 10044030, 10044031, 10044055, 10045271, 10055356, 10054692, 10047700 |
| General disorders and administration site conditions | 10008531, 10011912, 10011914, 10061818, 10014222, 10050068, 10058720, 10016059, 10016256, 10016558, 10016791, 10017577, 10018065, 10018112, 10021113, 10064774, 10022095, 10062466, 10025482, 10028154, 10054482, 10062501, 10033371, 10042435, 10069480 |
| Infections and infestations | 10056519, 10061640, 10003011, 10003012, 10065744, 10003999, 10061695, 10005047, 10061017, 10006259, 10055078, 10007810, 10065761, 10008330, 10010741, 10010742, 10061788, 10065765, 10058666, 10064687, 10065752, 10014594, 10014621, 10014678, 10014801, 10058838, 10015109, 10058804, 10015929, 10016936, 10017544, 10062632, 10018784, 10056522, 10058827, 10019799, 10080137, 10021881, 10021918, 10023216, 10023424, 10023874, 10065755, 10061229, 10050823, 10057483, 10027199, 10065764, 10028524, 10061304, 10033072, 10033078, 10055005, 10051741, 10069138, 10034016, 10058674, 10061912, 10051472, 10065766, 10057262, 10034835, 10056627, 10061351, 10050662, 10037888, 10059827, 10039413, 10062156, 10040047, 10040555, 10040753, 10040872, 10065771, 10062255, 10062112, 10064505, 10043649, 10048762, 10044302, 10046300, 10052298, 10046571, 10062233, 10046914, 10054688, 10065772, 10048038 |
| Metabolism and nutrition disorders | 10000486, 10001598, 10001680, 10002646, 10012174, 10052426, 10020587, 10020639, 10020647, 10020667, 10020670, 10020680, 10020712, 10020870, 10020907, 10020943, 10020949, 10021005, 10021018, 10021028, 10021038, 10021059, 10065973, 10027433, 10029883, 10045152 |
| Musculoskeletal and connective tissue disorders | 10065775, 10003239, 10003246, 10066480, 10003988, 10006002, 10048677, 10048831, 10008496, 10015688, 10065799, 10016750, 10062572, 10018761, 10065779, 10023215, 10048706, 10065796, 10065800, 10023509, 10024842, 10028294, 10065776, 10065795, 10065895, 10028395, 10065783, 10028411, 10028653, 10028836, 10065781, 10031264, 10064658, 10031282, 10033425, 10065793, 10039020, 10039226, 10039722, 10065777, 10065778, 10065798, 10044684, 10065738 |
| Neoplasms benign, malignant and unspecified (incl cysts and polyps) | 10048293, 10028533, 10029104, 10040907, 10049737, 10055351, 10045158 |
| Nervous system disorders | 10053662, 10060929, 10000521, 10001540, 10001949, 10002653, 10002953, 10003074, 10003591, 10065417, 10065784, 10008164, 10009845, 10010250, 10012373, 10013573, 10013887, 10062872, 10013911, 10013951, 10014217, 10014625, 10015832, 10051272, 10061457, 10061185, 10018767, 10019211, 10020508, 10020765, 10061212, 10022763, 10023030, 10024264, 10024382, 10027175, 10027198, 10028041, 10065780, 10065794, 10028417, 10029205, 10029223, 10029864, 10053661, 10056388, 10033987, 10034580, 10034620, 10056238, 10036653, 10063636, 10061928, 10038130, 10063761, 10039906, 10041349, 10041416, 10041549, 10042244, 10042772, 10043245, 10044391, 10044565, 10060890, 10074765, 10061403, 10047166 |
| Psychiatric disorders | 10001497, 10002652, 10002855, 10010300, 10057066, 10012218, 10012260, 10012378, 10015533, 10019077, 10022437, 10022998, 10024419, 10024421, 10026749, 10034719, 10037175, 10037234, 10038743, 10042458, 10042464 |
| Renal and urinary disorders | 10069339, 10063575, 10048994, 10064848, 10063057, 10013990, 10068405, 10019450, 10019489, 10029164, 10037032, 10038359, 10038385, 10038419, 10038463, 10065368, 10046539, 10046543, 10046555, 10061574, 10062225, 10046593, 10046628 |
| Respiratory, thoracic and mediastinal disorders | 10001409, 10001723, 10002972, 10003504, 10003598, 10006437, 10006440, 10063524, 10053481, 10065746, 10006482, 10051228, 10011224, 10013963, 10015090, 10020039, 10020201, 10021143, 10023838, 10065786, 10065759, 10065735, 10065880, 10059639, 10023862, 10062667, 10023891, 10056356, 10028735, 10068319, 10034825, 10055315, 10065881, 10065706, 10050028, 10034844, 10035598, 10055319, 10035623, 10035742, 10035759, 10036402, 10036790, 10037375, 10037383, 10065873, 10037400, 10038695, 10038738, 10038921, 10039100, 10062244, 10040747, 10040975, 10041232, 10041367, 10042241, 10065787, 10065900, 10050816, 10047681, 10047924 |
| Skin and subcutaneous tissue disorders | 10001760, 10005901, 10006556, 10013786, 10014184, 10015218, 10015277, 10016241, 10055525, 10019049, 10020112, 10020642, 10020649, 10020864, 10021013, 10062315, 10028689, 10028691, 10049281, 10062283, 10033474, 10054524, 10034966, 10037087, 10037549, 10037847, 10037868, 10049120, 10040785, 10040799, 10040865, 10040868, 10051837, 10040947, 10042033, 10042344, 10043189, 10044223, 10046735 |
| Vascular disorders | 10073529, 10007196, 10016825, 10019428, 10020407, 10020772, 10021097, 10065773, 10025233, 10048642, 10034578, 10034879, 10042554, 10042569, 10043565, 10047065, 10047115 |
